# *Salmonella* Virulence and Immune Escape

**DOI:** 10.3390/microorganisms8030407

**Published:** 2020-03-13

**Authors:** Mengyao Wang, Izhar Hyder Qazi, Linli Wang, Guangbin Zhou, Hongbing Han

**Affiliations:** 1 Beijing Key Laboratory for Animal Genetic Improvement, College of Animal Science and Technology, China Agricultural University, Beijing 100193, China; 13836985140@163.com (M.W.); 15870619927@163.com (L.W.); 2 Key Laboratory of Animal Genetics and Breeding of the Ministry of Agriculture, College of Animal Science and Technology, China Agricultural University, Beijing 100193, China; 3 Farm Animal Genetic Resources Exploration and Innovation Key Laboratory of Sichuan Province, College of Animal Science and Technology, Sichuan Agricultural University, Chengdu 611130, China; vetdr_izhar@yahoo.com; 4 Department of Veterinary Anatomy and Histology, Shaheed Benazir Bhutto University of Veterinary and Animal Sciences, Sakrand 67210, Pakistan

**Keywords:** *Salmonella*, virulence, immune escape, immune response

## Abstract

*Salmonella* genus represents the most common foodborne pathogens causing morbidity, mortality, and burden of disease in all regions of the world. The introduction of antimicrobial agents and *Salmonella*-specific phages has been considered as an effective intervention strategy to reduce *Salmonella* contamination. However, data from the United States, European countries, and low- and middle-income countries indicate that *Salmonella* cases are still a commonly encountered cause of bacterial foodborne diseases globally. The control programs have not been successful and even led to the emergence of some multidrug-resistant *Salmonella* strains. It is known that the host immune system is able to effectively prevent microbial invasion and eliminate microorganisms. However, *Salmonella* has evolved mechanisms of resisting host physical barriers and inhibiting subsequent activation of immune response through their virulence factors. There has been a high interest in understanding how *Salmonella* interacts with the host. Therefore, in the present review, we characterize the functions of *Salmonella* virulence genes and particularly focus on the mechanisms of immune escape in light of evidence from the emerging mainstream literature.

## 1. Introduction

*Salmonella* is a flagellated rod-shaped Gram-negative facultative anaerobe which infects multiple animal hosts including humans by contaminating a wide variety of foods [1,2,3,4]. *Salmonella enterica* (*S. enterica*) is regarded as the most pathogenic species and includes > 2600 serovars characterized until now [5]. With regard to human diseases, *Salmonella* is divided into two groups: Typhoidal serotypes and thousands of non-typhoidal *Salmonella* serotypes (NTS). Typhoidal serovars causing typhoid fever include *Salmonella enterica* serovar Typhi (*S*. Typhi), Paratyphi (*S*. Paratyphi), and Sendai (*S*. Sendai) [6,7]. The most common NTS are serovars Typhimurium (*S*. Typhimurium), Enteritidis (*S*. Enteritidis), and Dublin (*S*. Dublin) [8]. Infection with NTS ordinarily results in gastroenteritis, diarrhea, and fever (almost always present), with a low case fatality [9,10]. In addition to diarrheal disease, non-typhoidal *Salmonella* infections can invade normally sterile sites, resulting in bacteremia, meningitis, and other focal infections [11,12]. The invasive non-typhoidal *Salmonella* (iNTS) disease is usually characterized by the presence of the nonspecific fever similar to malaria and other febrile illnesses, resulting in clinically indistinguishable and higher case fatality than the non-invasive infections [11,13]. Different serotypes of *Salmonella* have different hosts, food sources, and pathogenesis, making their control challenging and complicated serotypes [14,15].

*S*. Typhi, *S*. Paratyphi, and *S*. Sendai are all human restricted [16,17,18]. Following ingestion and overcoming the resident microbiota, *Salmonella* initially colonizes the distal part of the small intestine [19]. Typhoidal *Salmonella* (TS) possesses specific virulence factors including typhoid toxin and virulence capsular polysaccharide (Vi antigen) that are involved in the development of symptoms and immune evasion [20,21]. The bacteria invade the intestinal mucosa, potentially through microfold (M) cells, and disseminate to the lymphatics and blood stream via phagocytes and ultimately spread to the spleen and liver [22,23,24]. These pathogens are invasive but do not normally trigger a rapid inflammatory response. Following recovery, some of the infected individuals are likely to become chronic carriers [25]. Typhoid infections are traditionally treated with ampicillin, chloramphenicol, and fluoroquinolones. However, physicians began moving away from commonly prescribed antibiotics due to an increased prevalence of multidrug-resistant (MDR) strains of *S*. Typhi. The transfer of antimicrobial resistance (AMR) genes between bacteria is commonly facilitated by plasmid or transposon exchange [26]. The AMR genes are generally associated with an IncHI1 plasmid which harbors a composite transposon that can carry multiple resistance genes [26,27].

Unlike TS, NTS has a broad host range. The infections caused by NTS are usually self-limiting and do not proceed beyond the lamina propria, but some iNTS have evolved a number of virulence genes which allow them to invade the intestinal mucosa and proliferate in phagocytes [28,29,30,31,32]. Both NTS and TS rely on two *Salmonella* pathogenicity islands (SPI) encoded type III secretion systems (T3SS), i.e., T3SS1 and T3SS2, which are essential for *Salmonella* invasion and dissemination [33]. Shortly after invasion, bacteria spread to systemic sites and cause systemic infection [34]. Fluoroquinolones, chloramphenicol, and oxytetracycline are commonly used to treat NTS infections. NTS develop the drug resistance by plasmids or integrons for destroying the activity of antibacterial drugs [35,36]. Point mutations within certain genes in *S*. Typhimurium have been identified as a potential cause of drug resistance [37]. Thus, prevention efforts are needed to reduce an unnecessary antimicrobial use in patient care settings and in food animals to help prevent the emergence of the resistance and infections with resistant NTS.

Difficulty in treating *Salmonella* infections is gradually increasing, and it has now become necessary to develop new treatment strategies. Vaccine development is a potential prospect for *Salmonella* control. This is particularly relevant given that the few licensed vaccines so far have targeted *S*. Typhi in people [38]. In essence, the ability to survive and replicate within the host phagocytes largely determines whether *Salmonella* can disseminate from the colonization site (intestines) to establish a systemic infection. Therefore, focused studies on how *Salmonella* escapes from host immunity and survives longer periods in short-lived and mobile myeloid cells will add a great value to our understanding of the host-pathogen interactions. In this review, by focusing on enticing findings of past and present studies, we briefly describe the mechanisms used by *Salmonella* to escape the innate and specific immunity to disseminate and establish infections.

## 2. Origin, Classification, and Diseases Caused by *Salmonella*

Since the first Kauffmann-White serotype scheme based on surface molecular antigen variation was published in 1934, serotyping has become the most important tool for identifying and classifying the *Salmonella* strains for more than 80 years [39,40]. Since 120 to 160 million years, *Salmonella* has evolved into a complex group of phenotypically diverse serovars. More than 2600 serotypes have been discovered since 1885 alone, resulting in antigenically distinct variants which are pathogenic in more than 100 species including mammals, birds, reptiles, and insects [5,41]. *Salmonella* genus is divided into two species, i.e., *S. enterica* and *Salmonella bongori* [42,43,44]. *S. enterica* is further classified into seven subspecies, i.e., *enterica* (I), *salamae* (II), *arizonae* (IIIa), *diarizonae* (IIIb), *indica* (IV), *houtenae* (VI), and several serovars previously assigned to the group IV (VII) based on biochemical and genomic modifications [41]. These subspecies are further classified into more than 50 serotypes based on O (somatic) antigen, and into multiple serotypes based on H (flagella) antigens [45]. Intriguingly, strains belonging to the subspecies I cause 99% of human cases of salmonellosis [46,47]. Meanwhile, *S. enterica* subspecies II, IIIa, IIIb, IV, VI, and *S. bongori* are usually isolated from cold-blooded animal species and environments but rarely from humans [41]. Recently, it has been proposed that high-throughput DNA sequencing can open a gateway to *Salmonella* serotyping and can improve our understanding regarding strains of public health relevance [48]. Whole-genome and metagenome sequence data permit the continuation of traditional serovar nomenclature and enhance the ability to infer true phylogenetic relationships between isolates [49].

The major clinical syndromes caused by *Salmonella* infection in humans are divided into typhoid fever that is predominantly caused by *S.* Typhi, *S*. Paratyphi, and *S*. Sendai [6,7,8], and a range of clinical syndromes including diarrheal disease caused by NTS. Typhoid is a human-restricted and highly adapted invasive disease, while NTS has a wide range of vertebrate hosts and more severe and invasive presentation in immunocompromised adults [50].

Typhoid fever remains a predominant enteric fever worldwide; meanwhile, an increasing incidence of enteric fever caused by *S*. Paratyphi A is also reported [6,51]. A principle difference between *S.* Typhi and other strains is the presence of Vi antigen. The Vi antigen is considered to be a virulence factor of *S.* Typhi, which modulates the different pro-inflammatory signaling pathways and allows *S*. Typhi to survive and replicate in the host cells, particularly the phagocytes [52]. *S.* Typhi uses these cells to disseminate to systemic sites of the body, such as the liver, spleen, and bone marrow. It is estimated that 5% of infected individuals will not be able to clear the infection within one year and enter a chronic carrier state where bacteria mainly reside in hepatobiliary tract and gallbladder, and thus increase the risk of cancer development [25,53,54].

NTS is an acute gastroenteritis typically acquired orally through contaminated water, fruits, seafood, vegetables, and meat, especially poultry. Following ingestion through contaminated food or water, its incubation period can vary from 4 to 72 h, and acute symptoms, such as fever, chills, nausea and vomiting, abdominal cramps, and diarrhea can be observed [55,56]. Available data demonstrate that there are estimated 1.3 billion cases of gastroenteritis caused by *Salmonella*, leading to approximately three million deaths worldwide per year [57,58]. Due to the lack of clean water supplies and proper sanitation, mortality caused by NTS gastroenteritis is mainly observed in the developing countries, but it is also of a considerable importance in the developed countries [58,59]. There are hundreds of NTS serovars that may cause invasive NTS human disease with a varying invasive virulence [60,61]. iNTS disease is caused mainly by *S.* Typhimurium, *S.* Enteritidis, and *S.* Dublin [8]. The iNTS disease burden in Africa is especially caused due to urbanization with large populations living in crowded and insanitary conditions with poor access to potable water [62,63,64]. iNTS is more common amongst people with an impaired immunity, and typically represents a febrile systemic illness and lower respiratory tract disease, commonly attributable to co-infections with HIV or malaria [50,60,65,66].

## 3. The Virulence-Related Genes of SPI

**Figure 1:**
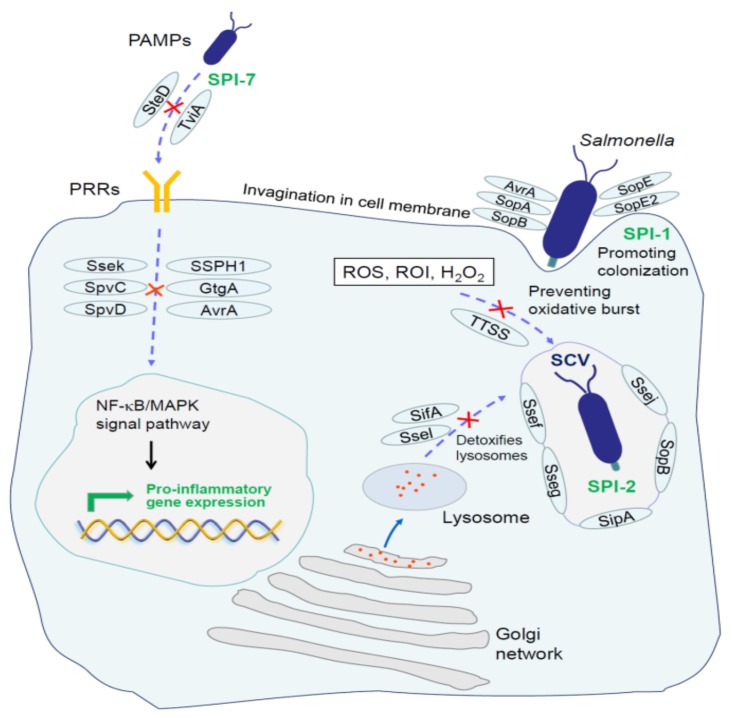
Role of *Salmonella* T3SS effectors in epithelial cells and macrophages. SopB, SopD, SopE, and AvrA are essential for membrane invasion during *Salmonella* infections. SipA, SseJ, SopE2, and SopB are required for biogenesis and correct localization of SCV. SifA and SopD2 contribute to evasion of lysosomal degradation. SPI-7 effector TviA is mainly responsible for masking the surface antigens, leading to the failure of PRRs to recognize them. Several effectors including SseL, GtgA, GogA, PipA, SpvC, and SpvD inhibit the innate immune signaling, and subsequently diminish the production of proinflammatory mediators and result in an inefficient clearance of phagocytized bacteria. *Salmonella* can also prevent the interaction of NADPH oxidase subunit Cytb558 with SCV and escape from the oxidative burst depends on T3SS. PAMP: Pathogen-associated molecular pattern; PRR: Pattern recognition receptor; ROS: Reactive oxygen species; ROI: Reactive oxygen intermediates; SCV: *Salmonella*-containing vacuole; SPI: *Salmonella* pathogenicity islands; NF-κB: Nuclear factor kappa beta; MAPK: Mitogen-activated protein kinase.

The specific regions encoding the virulence-related genes distributed in a cluster of *Salmonella* chromosomes and plasmids are called SPI. To date, 23 SPIs have been identified and characterized [33,67,68]. The five of these, i.e., SPIs-1–5 are common to all serotypes of *Salmonella* [69,70,71,72,73,74], whereas SPIs-19–23 are absent in both *S*. Typhi and *S*. Typhimurium, and are only present in a few *S. enterica* serovars including Dublin, Gallinarum, and Derby [67,68], and hence will not be discussed here. From SPIs-1–18, only SPI-1, 4, 9, 14, and 18 encoded effectors play an important role in *Salmonella* invasion into macrophages and epithelial cells. The virulence effectors secreted by SPI-2, 3, 5–8, 10–13, and 16 are implicated in helping *Salmonella* withstand the acidic environment, accomplishing the intracellular replication, and immune escape from the host. SPI-1 and SPI-2 contain a large number of virulence genes associated with the intracellular pathogenesis and co-encode T3SS, a molecular syringe which transfers the effectors from the bacteria into the host cell cytoplasm, and in turn, the effector manipulates allowing for bacterial invasion and replication in the host cells [75,76,77,78]. To date, over 40 SPI-1 and SPI-2 effectors have been identified in *S*. Typhimurium, *S*. Typhi, and *S*. Paratyphi A (Table 1) [17]. All serovars seem to have a set of “core” effectors, suggesting that they are critical for virulence in different hosts (PipA, PipB, PipB2, SifA, SipA, SipB, SipC, SipD, SopB, SopD, SpiC, SptP, SseF, SseG, SseL, SteA, and SteD). These effectors play diverse roles during infection. In Table 1, we have summarized the major effectors encoded by SPI-1 and SPI-2 in *S*. Typhimurium, *S*. Typhi, and *S*. Paratyphi A, and their functions. Among these 41 effectors identified in *S*. Typhimurium, 16 are absent in *S*. Typhi and *S*. Paratyphi A (AvrA, GogA, GogB, GtgA, GtgE, SlrP, SopA, SopD2, SpvB, SpvC, SpvD, SrfJ, SsaJ, SseJ, SseK2 and SsrA) [17]. Most of these effectors are from SPI-2, indicating that the role of SPI-2 in typhoidal serovars deserves further investigation. This may be related to the broad host range lifestyle of NTS and reflect the host restriction of TS. SopE2, SifB, SsaV, SseB, and SspH2 have a special mention, as they are present in *S.* Typhi but absent in *S*. Paratyphi A (Table 1) [17]. These dissimilarities in typhoidal strains may reflect differences between human restricted lifestyle of *S*. Typhi and *S*. Paratyphi. SspH2 promotes the colonization of *Salmonella* in host cells [79]. SopE2 is shown to contribute to generation of a replicative endosomal compartment in enterocytes and facilitate enterocytes invasion in vivo [80]. SpvB interferes with host intracellular iron metabolism via downregulation of nuclear factor erythroid-derived 2-related factor 2 (NRF2) [81]. SifB, a member of *Salmonella*-induced filaments (SIFs), is necessary for SIF formation and maintaining the integrity of *Salmonella*-containing vacuoles (SCVs) [82]. However, the potential function of SifB is still unknown [83]. The details regarding effectors are shown in Table 1 and Figure 1.

SPI-1 is a DNA fragment of around 40 kb with stable genetic traits, and present in all *Salmonella*. SPI-1 contains the *inv, hil, org, spt, spa, sip, iag, iac, prg, sic*, and other genes, encoding the regulator and secretory effector proteins of T3SS1. It is worthwhile to mention that not all genes within SPI-1 are associated with the T3SS1, but it has now been demonstrated that at least 29 T3SS1 genes are involved in different encoding functions. The regulators and effectors of T3SS1 are related to *Salmonella* colonization and invasion into intestinal epithelial cells and lead to necrosis and inflammatory reactions in macrophages [77,109]. Furthermore, these effectors are implicated in regulating the host cell exocytosis, interfering with host signal transduction pathways, and allowing *Salmonella* localization, survival and proliferation inside the vacuoles [95,110,111]. In addition, four genes, i.e., *sit A*, *sit B*, *sit C*, and *sit E*, play an important role in full virulence [112].

SPI-2 contains more than 40 genes which constitute four operons. From these, *ssa* encodes the T3SS2 [101], *ssr* encodes a secretion system regulator [113], and *ssc* encodes a molecular chaperone [114,115]. SPI-2-related secretion system T3SS2 delivers more than 20 effectors through the vacuole membrane into the host cytosol [100], playing an essential role during the second stage of host invasion which controls the survival and replication of *Salmonella* in phagocytes and epithelial cells [116]. At the same time, it allows *Salmonella* to escape the bactericidal effects of macrophages, and plays an important regulatory role in the progression of systemic infection and intracellular pathogenesis [74,116].

SPI-3, involved in the survival of *Salmonella* in macrophages, is around 17 kb and contains 10 ORFs constituting six transcription units. The major virulence gene encoded by SPI-3, *mgtCB*, is a high-affinity Mg^2+^ uptake system which is required for adaptation to nutritional limitations of the intra-phagosomal habitat [117]. From these, SPI-3 has been implicated in mediating the survival of *Salmonella* in macrophages and low Mg^2+^ environments [71].

SPI-4 is a 27 kb region that encodes a type 1 secretion system (T1SS), contributing to the adhesion of *Salmonella* to epithelial cell surfaces [118,119]. The SPI-4-encoded T1SS consists of five proteins (SiiABCDF) and secretes the giant adhesin SiiE, which is the largest protein in *Salmonella*, resulting in membrane ruffle formation and uptake of *Salmonella* [120,121].

SPI-5 is approximately 7 kb and plays a vital role in enteropathogenicity [122]. It encodes at least five genes, i.e., *pipA, pipB, pipC*, *pipD*, and *sopB*. The encoded proteins are related to the intestinal mucosal fluid secretion and inflammatory responses, and are regulated by SPI-1 and SPI-2 T3SS [122,123]. Recent studies on *Salmonella* have identified additional pathogenicity islands, such as SPI-6-23 [33,124,125,126,127,128,129,130,131,132,133,134]. *S*. Typhimurium and *S*. Typhi genomes contain six common SPIs (SPIs-6, 9, 11, 12, 13, and 16). SPI-7, 8, 10, 15, 17, 18 were considered to be present in *S*. Typhi genome, but absent in *S*. Typhimurium. SPI-14 is specific to *S*. Typhimurium [33]. Identification of new islands has improved our understanding regarding the members of *Salmonella* and their pathogenicity.

SPI-6 is approximately 59 kb and encodes a type 6 secretion system (T6SS) [135]. SPI-6 T6SS contributes to intra-macrophage survival and successful establishment of *S. enterica* in host gut during infection [136]. The transcriptional repression of the SPI-6 T6SS core component *clpV* resulted in defective intra-macrophage survival, attenuated virulence, and diminished systemic dissemination [137].

SPI-7 is the largest genomic island around 134 kb in length and encodes important virulence genes, including the major Vi antigen and IV_B_ operon in serovars Typhi, Paratyphi C and some strains of serovar Dublin [130]. These genes benefit bacteria against phagocyte-mediated killing and modulating the innate immune responses [138].

SPI-8 is approximately 6.8 kb region located adjacent to the *pheV* tRNA gene and encodes a degenerate integrase, two bacteriocin pseudogenes, and intact genes encoding proteins conferring resistance to these bacteriocins [133]. It has been speculated that proteins encoded in SPI-8 could improve bacterial fitness of typhoid serovars in human gut, however, further focused studies are required to support this caveat [133,134].

SPI-9 is about 16 kb island and encodes the type I system that helps in modulation of bacterial adhesion to the epithelial cells similar to the SPI-4 [129]. SPI-10 is approximately 32.8 kb in length, containing the *sefB*, *sefC*, *sefR,* and *prpZ* genes, which are implicated in the regulation of chaperone protein on mycelia manipulators [125]. From these, *prpZ* has been implicated in promoting *S*. Typhi survival in human macrophages [139,140].

SPI-11 includes *pagC*, *pagD*, and *msgA*, which reportedly have important roles related to the survival of *S.* Typhi in macrophages [141,142]. RaoN, a small RNA encoded within SPI-11, has been shown to be necessary for survival under in vitro stress conditions and contributes to the growth of *S.* Typhimurium in macrophages [128]. SPI-12, located next to the *proL* tRNA gene, is approximately 15 kb in *S*. Typhimurium and 6.3 kb in *S*. Typhi [33]. Regulation of genes within SPI-12 is conducive to in vivo adaptability [127]. SPI-13 is a 19 kb gene cluster and contributes to the virulence of *Salmonella* [143]. Recent studies have shown that SPI-13 mediated d-glucuronic acid (DGA) and tyramine (TYR) metabolic pathways can afford nutritional fitness to *Salmonella* Enteritidis (*S.* Enteritidis) [143].

SPI-14 is approximately 9 kb and specific to *S*. Typhimurium. LoiA, a novel virulence-regulating protein encoded in SPI-14, has been shown to be induced under low oxygen conditions and can enhance the ability of *S*. Typhimurium to invade host epithelial cells [144,145]. SPI-15, 16, and 17 were identified by bioinformatics in 2006 [146]. Studies on these islands are still very limited. SPI-15 is 6.5 kb, inserted near *glyU* tRNA, and is only present in *S*. Typhi, and absent in *S*. Typhimurium [146]. SPI-16 is found in *S.* Typhimurium and *S.* Typhi as a 4.5 kb fragment inserted next to *argU* tRNA. It is required for intestinal persistence of *S*. Typhimurium in mice [147]. Comparatively, SPI-17 is 5.1 kb long, and inserted in *argW* tRNA encoding six open reading frames (ORFs) [146]. SPI-18 harbors two ORFs organized into an operon, *hlyE* and *taiA* genes, and both are implicated in virulence. TaiA is a novel invasin involved in an increased phagocytosis of *S*. Typhi by macrophages [148]. HlyE presents a complex regulation network which participates in different stages of infective process. It affects the Ca^2+^ homeostasis in epithelial cells by induction of slow, intracellular Ca^2+^ oscillations to control *S*. Typhi growth in cells [149].

## 4. Molecular Mechanisms of *Salmonella* Immune Escape

In healthy individuals, the host body can recognize and clear pathogens through the innate and acquired immunity by a strong host immune response. However, invasive *Salmonella* can evade the immune surveillance using the sophisticated strategies, and could replicate, survive, and cause the persistent bacterial infections in hosts without even exhibiting the typical clinical symptoms [150]. For example, it has been reported that, in certain cases, patients with typhoid fever may carry bacteria in their gallbladder for the rest of their lives [32]. In general, such infections do not show clinical symptoms, but are a potential threat to the host. These asymptomatic carriers presumably act as reservoirs for a diverse range of *S*. Typhi strains and may act as a breeding ground for new genotypes [25]. It has been reported that *S.* Typhi chronic infection facilitates the gallbladder cancer development in humans [151]. *S.* Typhimurium involved in the persistent infections is also difficult to eliminate, and infected patients often continue shedding these pathogens in the environment, resulting in disease transmission [25,32].

### 4.1. Escape of Innate Immune System

The innate immune system provides the first line of defense against invading microorganisms by inducing a variety of inflammatory and antimicrobial responses. It is also particularly important in the gastrointestinal tract, where *Salmonella* is first colonized, to resist against a large variety of pathogenic microorganisms. However, it is not surprising that *Salmonella* has evolved strategies to overcome and adapt to an inflammatory environment. Intestinal epithelial cells are a primary cellular barrier of the gut and critical for nutrient uptake [152]. The epithelial cells form a continuous intact physical epithelial barrier with interspersing tight junctions (TJs) between each cell. *Salmonella* may disrupt the TJs structure through SPI-1-secreted effectors resulting in an increased permeability to luminal antigens, degrading the mucosal barrier function [153]. Intestinal microflora play a crucial role in the host defense, and oral probiotics have been shown to increase intestinal antimicrobial activity and paneth cells, which are the main intestinal cells responsible for the production of immunoreactive antimicrobial peptide (AMP) [154]. This peptide helps stabilize the intestinal barrier, while promoting the stability of intestinal microbial flora. Musca domestica cecropin and JH-3 (an analog of hemoglobin peptide P3), as the novel AMPs, were recently found to have an obvious inhibitive effect on *S*. Typhimurium [155,156]. However, the presence of host AMPs activates the PbgA which is required to maintain PhoPQ system of *S.* Typhimurium, promoting remodeling of outer membrane and resistance to innate immune AMPs [157]. The transcytosis of *Salmonella* across the gut epithelium by M cells is important for the induction of efficient immune responses to mucosal antigens in the Peyer’s patches [158]. M cells function as the antigen-sampling cells, selectively transporting *Salmonella* antigens and delivering the latter to the underlying lymphoid tissues where protective immune responses are initiated [22,159]. Paradoxically, *Salmonella* exploit M cells as a route for the host invasion. Both *S*. Typhi [160] and *S*. Typhimurium [161] selectively target and invade M cells through SPI-1.

During *Salmonella* invasion of the host cells, its surface pathogen-associated molecular patterns (PAMPs) are recognized by the host cell pattern recognition receptors (PRRs) [152]. The PAMPs which are significantly expressed by *Salmonella* include: Lipoprotein, curli amyloid fibrils, lipopolysaccharide (LPS), flagellin, and CpG DNA, which are recognized by PRRs. In addition to identifying the PAMPs, PRRs can also recognize the “danger-associated molecular patterns” (DAMPs). During an invasive *Salmonella* infection, innate immune responses are initiated by PAMPs and DAMPs, leading to the activation and recruitment of neutrophils and macrophages.

Extensively studied PRRs include the Toll-like receptors (TLRs) and NOD-like receptors (NLRs) [162,163,164]. TLRs recognize *Salmonella* on the cell surface and in endosomes, whereas NLRs detect *Salmonella* components in the cytosol. In an early stage of *Salmonella* infection, recognition of ligands by TLRs increases the bactericidal activity of local tissue macrophages, induces the maturation and migration of dendritic cells, and initiates the production of inflammatory cytokines and chemokines [165]. Curli amyloid fibrils are recognized by the TLR2/TLR1 heterodimer complex. It was shown that inability to produce curli fibrils will markedly reduce the ability of HeLa cells to respond to stimulation with intact *S*. Typhimurium [166]. Moreover, epithelial cells augment the barrier function via recognizing *S*. Typhimurium curli fibers in the gut by activating TLR2/phosphatidylinositol 3-kinase (PI3K) pathway [167]. In addition to curli fibrils, intact *Salmonella* contain triacyl lipoproteins that also stimulate responses through the TLR2 receptor [166,168]. TLR4 directly recognizes LPS, one of the main components of *Salmonella’s* outer membrane, promotes proinflammatory cytokine production, and phagocytic cell recruitment [169]. It is known that LPS is not homogeneous [170]. Additionally, studies have found that structural and chain length differences in LPS between serotypes of *Salmonella* are sufficient to drive different host immune responses [171,172,173]. *S*. Typhimurium uses PbgA and PmrA/Pmrb system to influence LPS assembly and drive variable host Type I IFN responses for their survival in various ecological niches [157,173,174,175]. The flagellin and non-methylated CpG sequence in *Salmonella* DNA are easily recognized by TLR5 [176] and TLR9 [177,178], respectively. Following ligand binding, TLRs engage the signaling adaptors MyD88 and TRIF, which are recruited in the C-terminal domain of TLRs. This recruitment initiates the downstream signaling and subsequently induces the host cells to produce inflammatory factors (interleukin-8, interleukin-10, interferon-α, and others), causing an infiltration of neutrophils to the site of infection and thereby producing an inflammatory response [179]. However, it has been demonstrated that *S.* Typhi can prevent neutrophil recruitment in the intestinal mucosa by masking its surface antigens with SPI-7 and interfering with TLRs [138,180,181]. Moreover, a SPI-7-encoded regulatory protein TviA can reduce TLR5-mediated inflammatory responses by controlling capsule expression and flagellar movement (Figure 1) [182,183]. These evidences indicate that the encoding genes locus SPI-7 in *S.* Typhi is a necessary factor for escaping the host inflammatory reactions. Capsules in *S*. Typhimurium are wrapped around LPS, which also prevents the inflammatory response induced by TLR4 recognition [181]. Even if TLRs successfully identify the PAMPs, *Salmonella* SPI-2 encoded proteins, i.e., SseL, SpvD, PipA, GogA, GtgA, SpvC, can inhibit the nuclear factor kappa beta (NF-κB), extracellular signal-regulated kinase (Erk), and mitogen-activated protein kinase (MAPK) activation, thus suppressing the transcriptional responses leading to inflammation (Figure 1) [85,106,184,185,186]. SseL acts as a deubiquitinase and prevents the ubiquitination of IkB-α. It results in the inability of IκB-α to dissociate from NF-κB, leaving NF-κB in an inactive state [106]. SpvD targets the NF-κB pathway by interfering with nuclear translocation of p65 [184]. PipA, GogA, and GtgA redundantly target components of NF-κB signaling pathway to inhibit transcriptional responses leading to inflammation [86]. SpvC removes phosphate groups of Erk and p38 MAPKs by phosphothreonine lyase to interfere with the downstream signaling pathways [185,186]. SseK suppresses TNF-α-induced, but not TLR-induced NF-κB, activation and cell death during macrophage infection [105]. Moreover, the effector AvrA transcribed by SPI-1 is able to stabilize the intestinal epithelial permeability and tight junctions of intestinal epithelial cells to mitigate a destructive effect produced by other SPI-1 effectors (i.e., SopB, SopD, SopE, and SopE2) (Figure 1). It was shown that disintegration of tight junctions in the intestinal epithelial cells could enhance the intestinal inflammatory responses. Thus, *Salmonella* can also avoid the host inflammatory responses through AvrA [84].

Moreover, *Salmonella* can trigger their own phagocytosis by macrophages [187,188,189,190] and become encapsulated in SCV. The effector SipA, SseJ, SopE2, and SopB are required for biogenesis and correct localization of SCV [80]. SipA provides functional continuity between forced bacterial entry and the intracellular replicative niche by priming the SCV, and the localization of SseJ maintains the membrane integrity and stability of SCV [91,104]. SopB is essential for efficient cytosolic proliferation of *Salmonella* (Figure 1) [191]. Once *Salmonella* become established within SCV, they become hidden from many extracellular detection mechanisms. SseF and SseG anchor SCV at the Golgi network and remain in this region during first few rounds of bacterial replication, forming a clustered microcolony of vacuoles (Figure 1) [192]. However, macrophages have evolved NLRs that can recognize the presence of PAMPs in the cytosol [164]. Upon binding to the ligand, the NLRs initiate different signaling cascades. NOD1 and NOD2 interact with a common adaptor protein called receptor-interacting protein 2 (RIP2) to mediate an efficient clearance of *Salmonella* from mucosal tissue [193,194]. Inflammasome assembly is usually triggered by the cytosolic NLRs which sense dangerous signals. It consists of NLRs, the adaptor proteins apoptosis-associated speck-like protein containing a CARD (ASC) and the effector molecules caspase-1, resulting in caspase-dependent secretion of mature pro-inflammatory cytokines IL-1β, IL-18, and pyroptotic cell death [195,196]. Mouse NLR apoptosis inhibitory protein (NAIP2) and human NAIP can recognize the *S*. Typhimurium T3SS inner rod component PrgJ, and NAIP5 can recognize *S*. Typhimurium flagellin D0 domain to induce NLR family CARD-domain containing protein 4 (NLRC4) phosphorylation and caspase-1 activation [197,198,199]. SCV lysis releases bacterium into the macrophage cytosol, where it is detected by the noncanonical inflammasome and eventually induces the pyroptotic death of the host cell [200,201]. However, SPI-2-mediated T3SS2 secrets effectors into the cytoplasm, and these effectors protect against the harmful environmental factors by regulating the vacuoles and intracellular biochemical reactions to facilitate the survival and replication of *Salmonella* in SCV [82,83,86,87,89,92]. Studies have demonstrated that human macrophage death and IL-1β production are elicited by *S.* Typhimurium SPI-1 but suppressed by SPI-2 [87,202]. SPI-2 supports the SPI-1-driven active infection of human macrophages and intra-macrophage bacterial survival [202].

Another potential reason that *Salmonella* induces its own phagocytosis by macrophages may be to avoid the phagocytic killing by neutrophils. This is also supported by the fact that *Salmonella* has a limited ability to resist the neutrophil-mediated bactericidal effects. Lysosomes in phagocytic cells contain a variety of hydrolases for combating bacteria. Evading lysozyme degradation is an important strategy for the survival of intracellular bacteria. It has been reported that SCV can fuse with lysosomes [203,204]. Interestingly, a *Salmonella* effector SifA, which is required to maintain the SCV membrane, has the ability to reduce the lysosomal enzyme activity (Figure 1) [90]. In addition to SifA, *Salmonella* also uses SopD2 to interfere with endosome-to-lysosome trafficking (Figure 1) [96]. Therefore, in order to efficiently kill pathogens in SCV, host cells are required to generate a stronger bactericidal environment.

**Figure 2:**
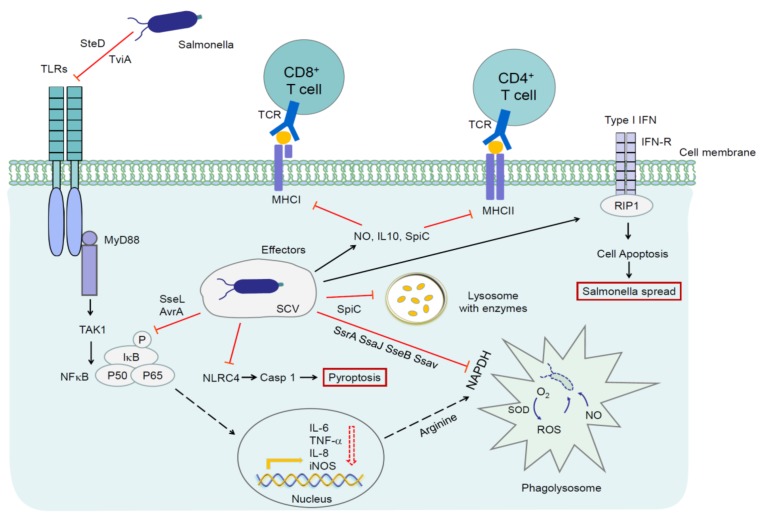
Mechanisms by which *Salmonella* escape host immune responses. SPI-2 effector SpiC prevents DCs from presenting antigens to MHCs, and SifA blocks MHC II expression, resulting in an inadequate activation of naive T cells. SPI-2 effector SteD with its chaperone SrcA is a key requirement for *Salmonella* which suppress T cell activation by forcing an inappropriate ubiquitination of MHC II. SseB, SsrA, SsaJ, Ssav are used for avoiding the oxidative burst. *Salmonella* also increases IL-10 and NO production and induces the expression of iNOS to inhibit the proliferation of T cells. Furthermore, when SPI2 is activated, the expression of flagellin in the intracellular environment is inhibited, preventing the NLRC4 from recognizing *Salmonella*. During the course of infection, *Salmonella* exploits the host type I interferon response to eliminate the macrophages through RIP-dependent cell death and promotes its own survival. TLR: Toll-like receptor; MyD88: Myeloid differentiation primary response gene 88; TAK1: Transformed growth factor kinase 1; IκB-α: NF-κB inhibitor alpha; TCR: T cell receptor; MHC I: Major histocompatibility complex class I; MHC II: Major histocompatibility complex class II; NLRC4: NLR family CARD domain containing 4; CASP1: Caspase 1; IL-6: Interleukin 6; IL-8: Interleukin 8; IL-10: Interleukin 10; TNF-α: Tumor necrosis factor a; iNOS: Inducible nitric oxide synthase; IFN: Interferon; IFN-R: Interferon-a/b receptor; SOD: Superoxide dismutase; NADPH: Nicotinamide adenine dinucleotide phosphate; NO: nitric oxide; RIP: receptor-interacting protein.

Oxidative bursting catalyzed by nicotinamide adenine dinucleotide phosphate (NADPH) oxidase is induced by phagocytic cells to produce a large number of reactive oxygen intermediates (ROI), such as O_2_^-^ and H_2_O_2_, which are converted into a strong oxidant hypochloric acid and rapidly kill *Salmonella* [205]. However, *Salmonella* depends on SPI-2 effectors, i.e., SseB, SsrA, SsaJ, and Ssav for preventing an interaction of NADPH oxidase subunit Cytb558 with SCV to avoid the oxidative burst (Figure 2) [102,206]. In addition, *Salmonella* can resist the oxidative killing effect of ROI using catalase, antioxidant proteins, and superoxide dismutase [207]. Reactive nitrogen intermediates (RNI) include nitric oxide and its derivatives, such as nitrososulfur compounds, nitrogen peroxide, etc. Reactive nitrogen intermediates can also kill *Salmonella* through various mechanisms, such as by causing DNA damage, preventing SPI-2 transcription, and inhibiting the PhoP/PhoQ acid-tolerance regulation reaction [208]. However, *Salmonella* also possesses the NO_2-_ operating system and nitrate reductase for protection against RNI damage [209].

Eswarappa and colleagues have shown that most SCVs in macrophages contain only one bacterium. *Salmonella* replicates in the SCV, and with an increasing bacterial number, one SCV divides into multiple SCVs, which benefits the survival of the intracellular bacteria [210]. On the one hand, it becomes much more difficult for the host cells to combat multiple SCVs compared to a single SCV, as this effort also requires more bactericidal media. In addition, a bacterium occupying a single SCV reduces the competition for nutrients and secretes effectors more efficiently into the cytoplasm [210]. Macrophages provide a safe haven to *Salmonella* for its survival and proliferation. However, when nutrients in the host cells are depleted, *Salmonella* is forced to induce the host cell death and search for new a host instead. *Salmonella* mediates macrophage death through two mechanisms [211]. One of these mechanisms involves the rapid induction of macrophage death. *Salmonella* expressing the SPI-1 T3SS rapidly trigger caspase-1-dependent apoptosis of infected macrophages [94,212,213]. Murine bone marrow-derived macrophages undergo lysis within 1 h of infection [214]. This rapid activation of programmed macrophage cell death depends on SPI-1 encoded protein SipB, bacterial flagellin, and the T3SS1 export machinery [211,215]. However, the other mechanism is SPI-1-independent, and characterized by a delayed induction of apoptosis to kill infected macrophages as late as 18 h post-infection. A functional T3SS2 and *OmpR* (ancestral regulator involved in the expression of ssrAB operon located in SPI-2) are required for the delayed induction of apoptosis, and allow *Salmonella* to spread intercellularly within apoptotic bodies [211,216,217]. Furthermore, past studies have indicated that the rapid and delayed activations of programmed macrophage cell death are independent of each other, since the mutations in SPI-1 do not affect the delayed induction of apoptosis, and the mutations in SPI-2 do not affect rapid induction of apoptosis [211]. The dead or dying macrophages containing *Salmonella* are engulfed by other macrophages recruited at the site of infection, and these macrophages can again serve as a safe haven for *Salmonella* to survive, while avoiding the extracellular host defenses [218]. Furthermore, studies have revealed that there are subpopulations of vacuolar and cytosolic *Salmonella* [219]. Vacuolar *Salmonella* are T3SS2-induced, whereas cytosolic *Salmonella* are induced by T3SS1 and flagellated [220]. The release of bacteria from SCV leads to their transcriptional reprogramming and a robust replication in the cytosol that exceeds their replication rate in the SCV [220,221]. However, the permissiveness of *Salmonella* survival and replication following vacuole lysis is dependent upon the cell type. For instance, *Salmonella* eventually hyper-replicate in the cytosol of epithelial cells but not in the cytosol of fibroblasts or macrophages [222]. Epithelial cells infected with *Salmonella* trigger an acute intracellular amino acid starvation, resulting in the induction of xenophagy to protect the host cells from *Salmonella,* but it is temporally restricted and not absolute [219,223]. Eventually, epithelial cell death via pyroptosis results in cell lysis, proinflammatory cytokine release, and escape of the cytosolic bacteria into the extracellular space, providing a potential mechanism of dissemination [219].

### 4.2. Escape of Adaptive Immune Responses

As the antigen presenting cells, macrophages and dendritic cells (DCs) can directly recognize the PAMPs in bacteria and present the bacterial antigens to T cells, initiate the proliferation and differentiation of naive T cells into the effector T cells, playing an important role in adaptive immune response against the invading bacteria. Interference with these functions is likely to increase the survival chances and invasion of bacteria in the hosts. Therefore, it seems that manipulating the antigen presentation capability of antigen presenting cells is another important strategy of pathogens for suppressing and escaping the host immune responses [224]. SseI has been shown to block the migration of DCs to lymphocytes [225]. Moreover, the major histocompatibility complex (MHC) plays an important role in combating the *Salmonella* during the later stages of infection [226,227]. It was shown that in human cells harboring intracellular *Salmonella*, SPI-2-encoded SifA is responsible for interfering with major histocompatibility complex class II (MHC II) cell surface expression and thereby provides *Salmonella* with a specific mechanism to evade or delay the host adaptive immune response (Figure 2) [228]. SPI-2-encoded SteD with its chaperone SrcA can force an inappropriate ubiquitination of MHC II to suppress T cell activation (Figure 2) [229,230]. Tobar and colleagues have shown that *Salmonella* prevents the degradation of lysosomes in DCs by SPI-2 effector SpiC, making it impossible for DCs to bind and present antigens to MHC, thus preventing the differentiation of naive T cells [231] (Figure 2). Once T cells are activated during infection, the majority of both CD4+ and CD8+ T cells have acquired an activated phenotype and an unexpectedly large fraction of these T-cell populations secreted IFN-γ to inhibit bacterial replication [232,233,234,235]. IL-12 has been identified as a major IFN-γ inducer. Interestingly, persons lacking the IL-12 receptor are more susceptible to *Salmonella* infection [236,237]. TNF-α also controls *S*. Typhimurium replication levels in persistently infected hosts [234]. Despite a profound activation of both CD4+ and CD8+ populations, expansion of either T-cell population was marginal [238]. Only a moderate (two- to three-fold) expansion of these T-cell populations were observed over several weeks of infection. *Salmonella* induces the expression of inducible nitric oxide synthase (iNOS) by SPI-2 to inhibit the proliferation and differentiation of T cells [224]. Furthermore, in mice models, *S.* Typhimurium infection resulted in immunosuppression by increasing IL-10 and nitric oxide (NO) production with immunosuppressive activity [233,239,240,241] (Figure 2).

Both CD4^+^ and CD8^+^ T lymphocytes and the humoral immune responses are required to control *Salmonella* infection [242,243,244,245,246,247,248]. It has been demonstrated that mice continuously infected with *Salmonella* have higher antibody titers [232] including IgA, IgM, and IgG [232,249], indicating that B cells also play an important role in the host defense. This may represent a deliberate shift from Th1/Th17 to Th2 responses [250]. Moreover, the adaptive immune responses also provide a positive feedback to the innate immune system [251]. This feedback is mediated via cytokines synthesis, leading to an increased number and activation of effector cells, and subsequently producing an increased antimicrobial response.

## 5. Perspectives

In recent years, different disciplines, such as immunology, microbiology, and cell biology have contributed greatly to our understanding of the interaction between *Salmonella* and the host. Several studies have revealed complex interactions between microbial pathogens and higher organisms. Future studies will hopefully expand our understanding of an interplay between immunity and bacteria in different infected organs. At present, our understanding of the interaction of *Salmonella* with innate and adaptive immunity evading the host defense strategies in humans is still incomplete. When the effects of normal microbial colonization flora contained in the host and the diversity of environmental conditions are analyzed, the complexity of the interaction between bacteria and host becomes far greater than our current knowledge in this domain. Invasive diseases caused by *Salmonella* remain a major factor accounting for the severe death and morbidity rates worldwide. Therefore, the ongoing research focusing on the relationship between *Salmonella* and the host immunity has the desired potential to explicate complex questions related to the *Salmonella*–host interactions and improve the prevention and treatment strategies aimed at combating these infectious diseases in the near future.

## Figures and Tables

**Table 1:** The functions of SPI-1/2 effectors in *S*. Typhimurium, *S*. Typhi, and *S*. Paratyphi A.

Effectors	Pathogenicity Island	Function (s)	Key Reference (s)
AvrA	SPI-1/SPI-2	Stabilizes the intestinal epithelial permeability and tight junctions; cysteine protease; inhibits NF-κB signaling	[84]
GogA	SPI-2	Cleaves the subset of NF-κB subunits; inhibits NF-κB signaling	[85,86]
GogB	SPI-2	Inhibits NF-κB signaling	[85]
GtgA	SPI-2	Inhibits NF-κB signaling	[85]
GtgE	SPI-1/SPI-2	Promotes replication inside murine macrophages	[87]
PipA	SPI-2	Cleaves the subset of NF-κB subunits; inhibits NF-κB signaling	[85]
PipB	SPI-2	Targeted to SIFs	[88]
PipB2	SPI-2	Resists extraction by high salt, high pH; implicated in recruitment of kinesin-1 to SCV	[89]
SifA	SPI-2	Detoxifies lysosomes; subverts human NLRP3 and NLRC4 inflammasome; required for SCV membrane stability; SIF formation; contributes to T3SS1-independent inflammation	[90]
SifB	SPI-2	Targeted to SIFs	[82,83]
SipA	SPI-1	Enhances actin filament assembly; promotes proliferation of cytosolic *Salmonella*; disrupts tight junctions; SCV trafficking	[91]
SipB	SPI-1	Cholesterol-binding translocon component; triggers apoptosis via caspase-1 activation in macrophages and DCs	[82]
SipC	SPI-1	Translocon component: mediates effector molecule translocation; promotes actin polymerization and bundling	[92]
SipD	SPI-1	Translocon component	[93]
Slrp	SPI-1/SPI-2	Inhibits the release of IL-1β	[87]
SopA	SPI-1	A HECT-like E3 ubiquitin ligase	[94]
SopB	SPI-1	Modulates SCV trafficking; phosphoinositide phosphatase; involved in phagosomal closure; enhances RhoG activation; disrupts tight junctions; stimulates chloride secretion; prevents apoptosis through activation of Akt	[95]
SopD	SPI-1/SPI-2	SIF formation, prevents accumulation of Rab32 on SCV and SIFs	[87]
SopD2	SPI-2	Targeted to SIFs and late endosomes	[96]
SopE	SPI-1	Promotes colonization of *Salmonella*; induces remodeling of actin	[97]
SopE2	SPI-1	Guanine nucleotide exchange factor for Cdc42; promotes pro-inflammatory signaling	[80]
SpiC	SPI-2	Interferes with vesicular trafficking in host cells to prevent SCV-lysosome fusion	[92]
SptP	SPI-1	Rho GAP domain functions in downregulating host membrane ruffling after entry; tyrosine phosphatase domain acts on ACK; vimentin; and presumably other substrates	[98]
SpvB	SPI-2	Promotes macrophage apoptosis and P-body disassembly	[81,87]
SpvC	SPI-1/SPI-2	Inhibits MAPK signaling	[99]
SpvD	SPI-1/SPI-2	Inhibits NF-κB signaling	[87]
SrfJ	SPI-2	Responses to intracellular conditions	[100]
SsaJ	SPI-2	Prevents the phagocyte NADPH oxidase from trafficking toward SCVs	[101]
Ssav	SPI-2	Prevents the phagocyte NADPH oxidase from trafficking toward SCVs	[102]
SseB	SPI-2	Prevents the phagocyte NADPH oxidase from trafficking toward SCVs	[102]
SseF	SPI-2	Tethers SCV to the Golgi network; contributes to Sif formation; replication of *Salmonella* in SCV	[87,103]
SseG	SPI-2	Tethers SCV to the Golgi network; contributes to Sif formation; replication of *Salmonella* in SCV	[87,103]
SseJ	SPI-2	Acyl transferase; cholesterol esterification; SCV membrane dynamics	[87,104]
SseK1	SPI-2	Inhibits TNFα-stimulated NF-κB signaling	[105]
SseK2	SPI-2	Related effectors that inhibits NF-κB signaling	[105]
SseL	SPI-2	Inhibits autophagic clearance of cytosolic aggregates; induces late macrophage cell death; inhibits directional migration of macrophages and DCs	[106]
SspH2	SPI-2	An E3 ubiquitin ligase; activates NOD1 signaling	[79,87]
SsrA	SPI-2	Prevents the phagocyte NADPH oxidase from trafficking toward SCVs	[102]
SteA	SPI-1/SPI-2	SIF formation, vacuolar membrane partitioning	[107]
SteC	SPI-2	Induces assembly of F-actin meshwork around SCV	[108]
SteD	SPI-2	Inhibits antigen presentation and T cell activation	[17]

NF-κB: Nuclear factor kappa beta; SCV: *Salmonella*-containing vacuole; NLRP3: the NOD-like receptor family, pyrin domain containing 3; NLRC4: NLR-family CARD-containing protein 4; SIF: *Salmonella*-induced filament; T3SS1: type III secretion system 1; HECT: homologous to E6-AP carboxy terminus; GAP: GTPase-activating phosphatase; ACK: a Cdc42-associated tyrosine kinase; NADPH: nicotinamide adenine dinucleotide phosphate; TNFα: tumour necrosis factor alpha; DCs: dendritic cells; NOD1: nucleotide-binding oligomerisation domain 1.
